# Alveolar Mucosa-Switching Technique for Augmentation of the Attached Immobile Mucosa

**DOI:** 10.7759/cureus.74110

**Published:** 2024-11-20

**Authors:** Takashi Kawase, Kazuhiro Matsushita, Naoki Anbo, Aya Matsuda, Hidekazu Yamamoto

**Affiliations:** 1 Dentistry, Kamishihoro Dental Clinic, Hokkaido, JPN; 2 Center for Advanced Oral Medicine, Hokkaido University Hospital, Sapporo, JPN; 3 Vascular Biology and Molecular Pathology, Graduate School of Dental Medicine, Hokkaido University, Sapporo, JPN; 4 Oral Biochemistry and Molecular Biology, Graduate School of Dental Medicine, Hokkaido University, Sapporo, JPN

**Keywords:** alveolar mucosa, collagen fiber, dental implant, elastic fiber, free gingival graft, palatal mucosa

## Abstract

In cases with a highly positioned mucogingival junction, an apically positioned flap in combination with palatal-mucosa grafting is commonly performed to deepen the sulcus. However, we believe that immobilizing the mucosa is more important than merely deepening it; thus, we developed a simple method based on the characteristics of its components. Elastic fiber should be replaced by collagen fiber. An apically positioned flap is no longer required and mobile alveolar mucosa, including elastic fibers, is just excised. Palatal-mucosa graft, including collagen fibers, is then performed. Mobile mucosa is switched to immobile mucosa, and histological evaluation supports this change. At the recipient site, simple excision of the mobile mucosa without an apically positioned flap is innovative.

## Introduction

Palatal-mucosa grafting is commonly performed in cases with a shallow vestibule around dental implants in the mandibular molar region, although some controversies persist regarding its necessity [1-3]. The purpose of this procedure is not only to augment the keratinized mucosa around dental implants but also to immobilize the mucosa. In cases with a highly positioned mucogingival junction, where the mobile alveolar mucosa occupies a large portion of the buccal surface of the alveolar bone in the vertical direction, immobilization of the alveolar mucosa should be further considered. Regarding the histological characteristics of mobile mucosa, elastic fibers are crucial for flexibility and distensibility [4]. Thus, eliminating elastic fibers may be effective for mucosal immobilization. The alveolar mucosa is dissected from the periosteum, excised, and replaced with palatal keratinized mucosa. Although the method is similar to vestibuloplasty, it differs from vestibuloplasty using an apically positioned flap because further deepening of the vestibular sulcus is never needed, and an elevated mucosal flap is not required. The vestibular depth is acquired by the vertical length of the excised and donor mucosa. The non-keratinized mobile mucosa is switched to keratinized immobile mucosa. The vestibular depth is essentially gained, followed by keratinization. Herein, we report the technique of mucosal switching along with supporting histological findings. The aim of this study is to introduce a simple technique, which is based on histological findings.

## Technical report

**Figure 1:**
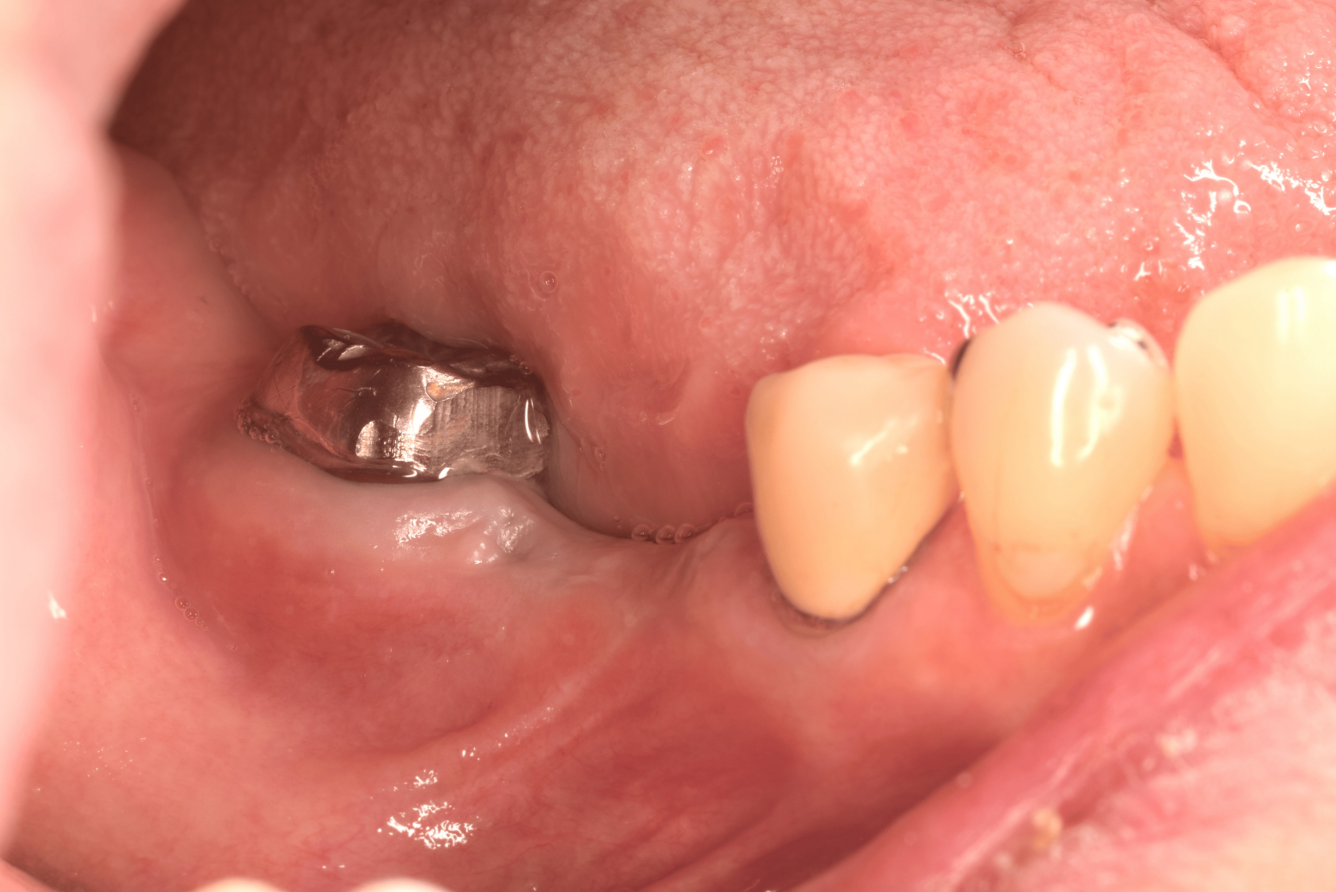
Mobile alveolar mucosa above the vestibular sulcus. A frenum is extended to the alveolar edge.

**Figure 2:**
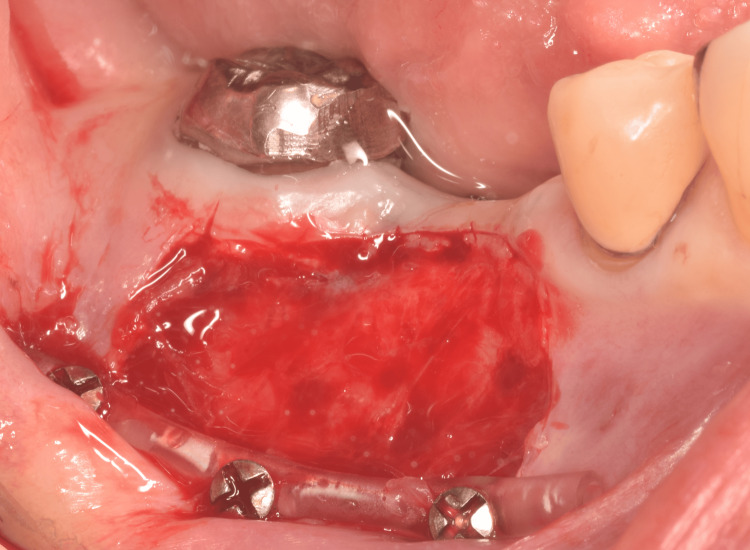
Preparation for the keratinized palatal-mucosa grafting. The mobile alveolar mucosa is dissected from the periosteum and excised. The surface of the periosteum is seen. Polyvinyl chloride 6Fr suction catheter tube is placed at the original vestibular position to avoid postoperative shrinkage of the vestibule and to enable easy suturing of the graft. Intentional deepening of the vestibular sulcus is not performed.

**Figure 3:**
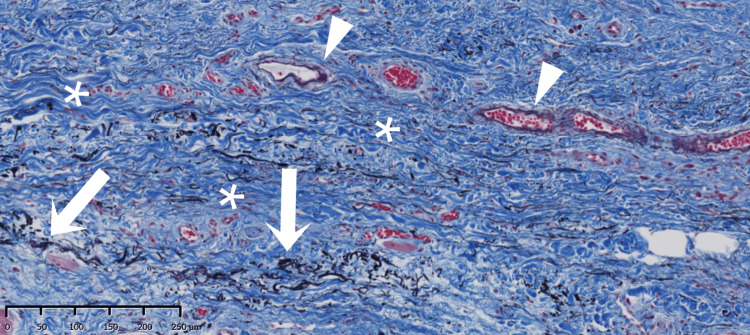
Histology of the excised mucosa. Collagen fibers (blue bundle: asterisk) are small and thin. Elastic fibers (seen as threadlike black fiber) are coexistent with collagen fibers (white arrow) and seen in the vessel wall (white arrowhead) (Elastica Masson staining).

**Figure 4:**
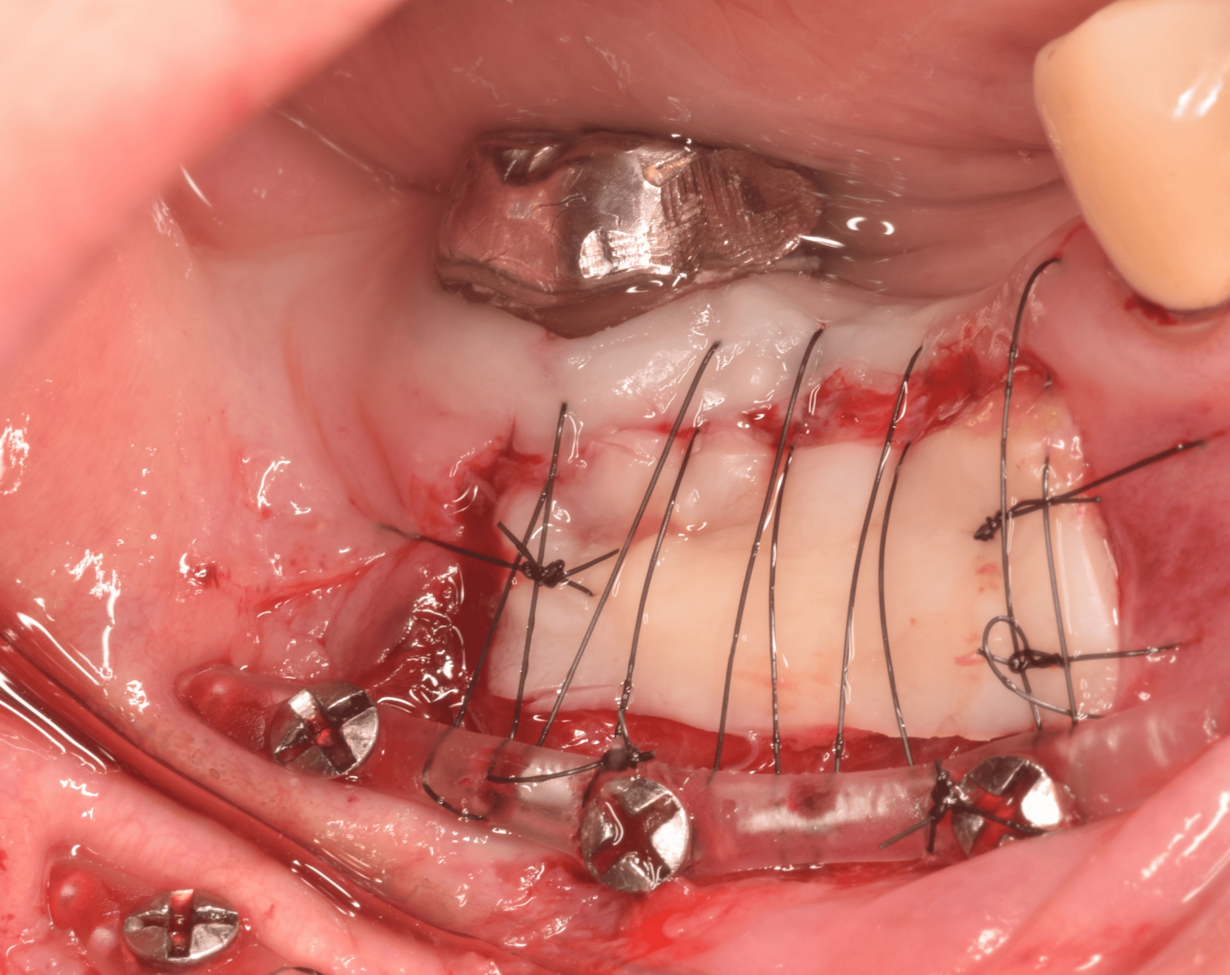
The local condition just after grafting of the palatal mucosa. A keratinized palatal graft is placed on the prepared periosteum and immobilized by horizontal mattress sutures. The sutures run in the inner lumen of the tube at the sulcus side and at the margin of the crystal side. A splint is placed over the graft for one week postoperatively.

**Figure 5:**
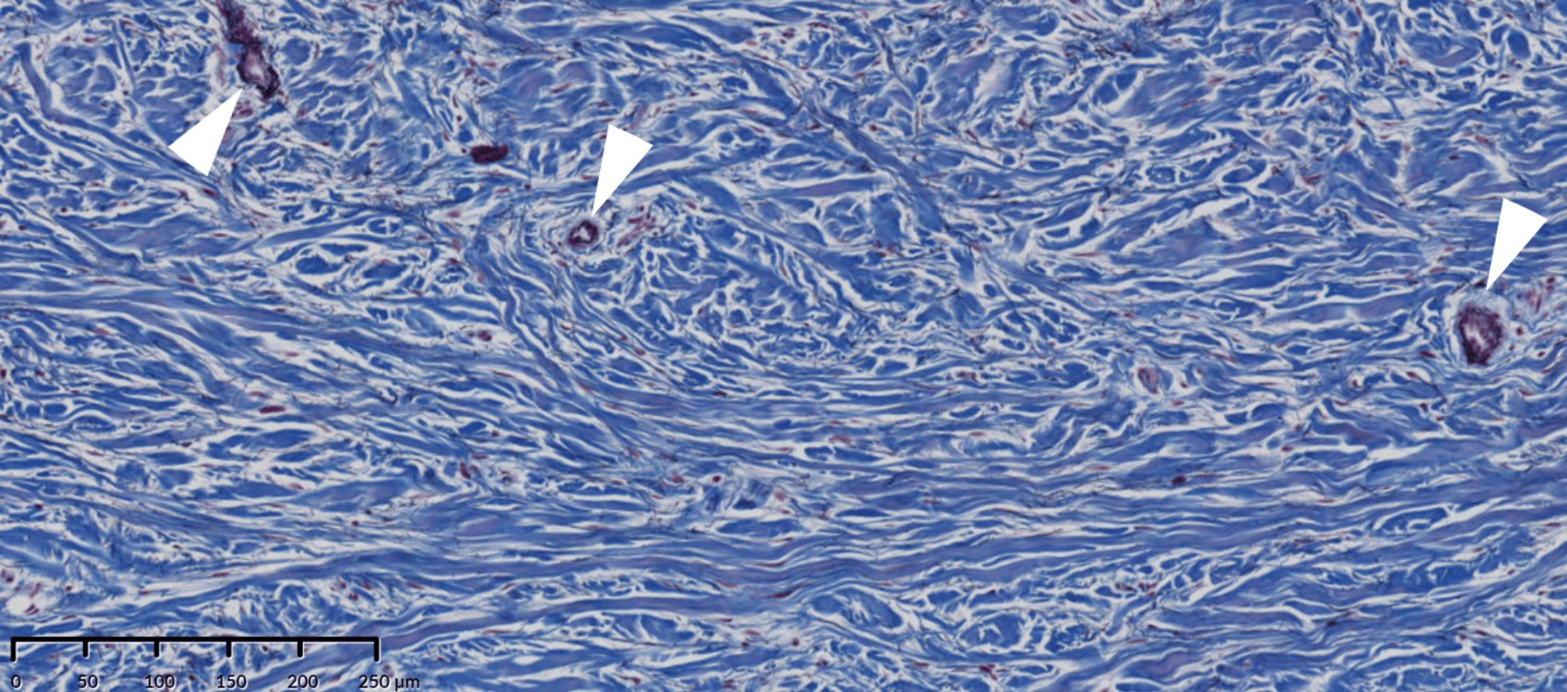
Histology of the grafted palatal mucosa. No elastic fibers are seen except in the vessel wall (white arrowhead). The connective tissue comprises sufficient collagen fibers (Elastica Masson staining).

**Figure 6:**
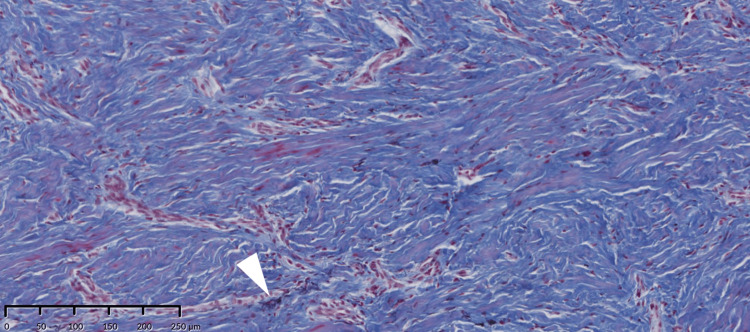
Sample taken from the alveolar recipient region at the second-stage surgery. No elastic fibers are observed except in the vessel wall (white arrowhead). The connective tissue structure at the recipient site reflects that of the donor site (Elastica Masson staining). The primal component of the alveolar mucosa is switched from elastic fiber to collagen fiber.

**Figure 7:**
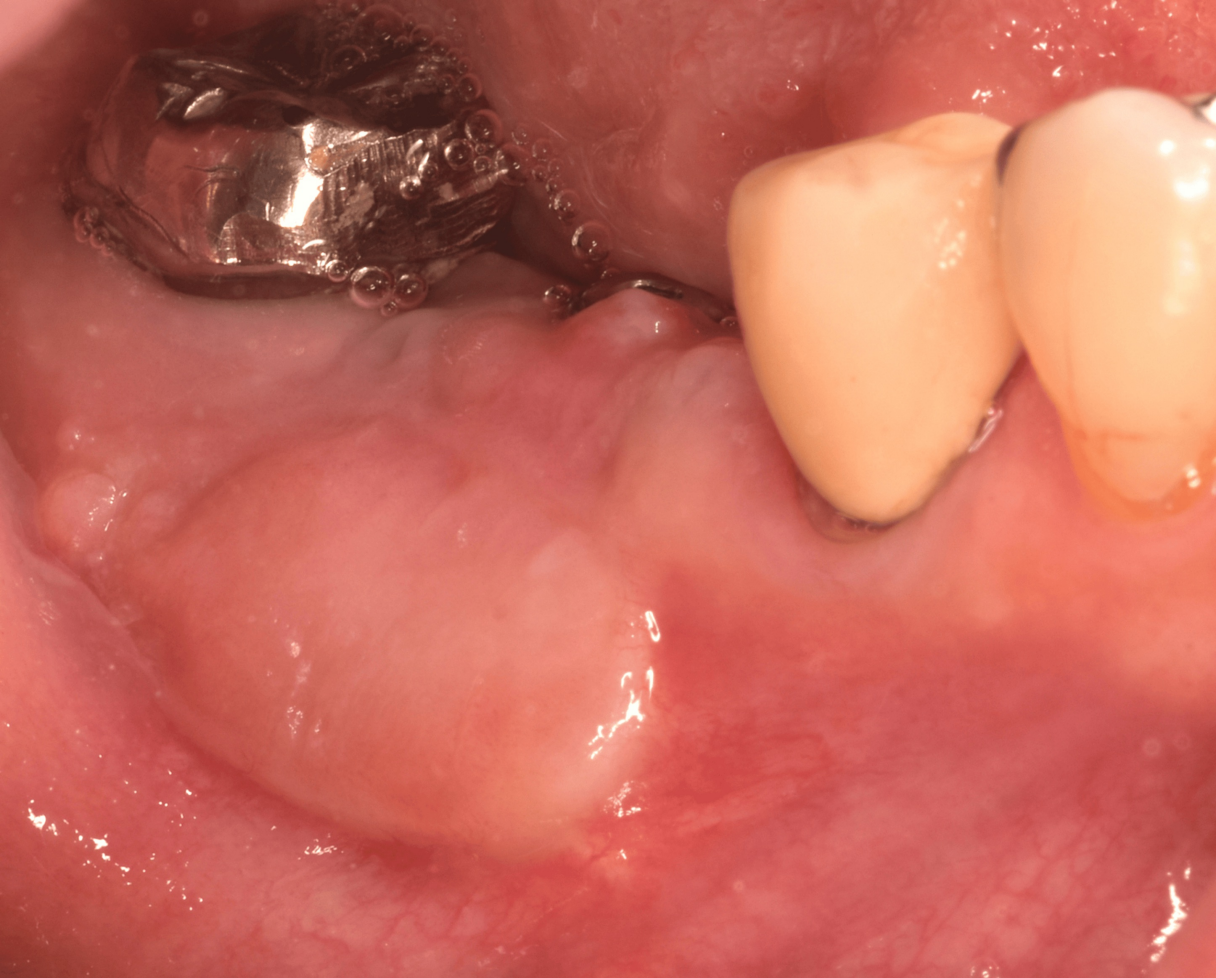
Final condition. The donor flap was well engrafted, and an immobile region was established at the buccal side. No flabby was observed at the inferior margin of the graft in the vestibule.

In the shallow molar region (Figure 1), a split-thickness flap of mobile alveolar mucosa was elevated according to the width and height required for immobilization between the mucogingival junction and the vestibule, and then excised, as it was no longer needed (Figure 2). Histological examination of the excised mucosa revealed relatively small bundles of collagen and elastic fibers scattered throughout the connective tissue (Figure 3). These characteristics facilitate elasticity. To avoid postoperative shrinkage of the vestibule due to regrowth of the alveolar mucosa from the lower margin in the vestibular sulcus, we performed our original method [5]. Briefly, continuous compression was applied using a polyvinyl chloride 6Fr suction catheter, which was fixed to the alveolar bone using Le Forte System screws (diameter 2.0 mm, length 6.0 mm; Jeil Medical Corp, Seoul, Korea) along the sulcus. It was performed before the excision of the mobile mucosa. The cortical bone screws passed through the inner lumen of the tube and apically positioned mucosa. For grafting, split-thickness immobile keratinized palatal mucosa was harvested from the canine-premolar area on the hard palate, at least 5 mm from the gingival margin, and placed on the periosteum where the mobile alveolar mucosal flap had been removed (Figure 4). The trimmed mucosal tissue at the edges was examined histologically. No elastic fibers were observed in the harvested flap, except those in the vessels (Figure 5). Periosteal stay sutures were used to stabilize the graft, and compression was applied using a holding splint for one week. The splint should never be removed in the meantime. Antibiotics and analgesics were prescribed for three days after surgery. Subsequently, to ensure secure engraftment, patients were instructed to wear the splint at all times for one month, except when washing the splint or rinsing the oral cavity after meals. Finally, splint, suction catheter, and screws were all removed. Satisfactory healing of the grafted flap was observed, and mobility was not observed. Two months later, a dental implant was placed at the top of the alveolar bone in a usual manner (first-stage surgery). Two months later, during the second-stage surgery for abutment construction, the surplus mucosa created around the healing cap and produced by mucosal adjustment was examined histologically. Elastic fibers were not observed except in the vessels (Figure 6). The mobile alveolar mucosa, which contained elastic fibers, was completely replaced by tissue-containing collagen fibers. Four years postoperatively, no visible contractions or gingival inflammation was observed (Figure 7). Switching the structural components resulted in reliable clinical outcomes.　

## Discussion

Apically positioned flap operation, followed by keratinized mucosal grafting, is commonly performed to increase the zone of attached mucosa and deepen the shallow vestibule [1]. However, the apically positioned flap will sometimes get flabby in the end and hygiene will be worse. We are sometimes skeptical regarding the necessity of the downward shift of mobile mucosa before grafting, and we came up with the idea that immobilization is rather important. In movable mucosa such as alveolar mucosa, elastic fibers are abundant components and play an important role in mobility [6,7]. On the other hand, in immobile gingiva such as palatal mucosa or attached gingiva, elastic fibers are almost absent, and collagen fibers are present in abundance [8]. Collagen fiber is indispensable for immobilization. Considering the above histological aspect, we tried the present method and achieved a fine postoperative condition. The number of elastic fibers decreased, and collagen fiber was replaced with it. As for the donor site, the property was different from location to location [9]. Although epithelial thickness does not differ between sites, lamina propria was thicker in the maxillary tuberosity than in the lateral palate. Our component of interest, type I collagen, is the predominant structural protein in the lamina propria. According to these properties, tuberosity is a more desirable donor site. However, it is not easily accessible because the second molar blocks the vision and blocks easy handling of the instruments as well. Then, we generally harvest the donor flap from the lateral palate. If the condition allows, harvesting from tuberosity should be taken into consideration for better outcomes.

The local condition has been quite stable, and no change has been found for more than four years. We believe that this phenomenon is due to the elimination of elastic fiber. The fibers are no longer required. The histological investigation confirmed this finding. There have been no reports on methods to improve mobile mucosa through simple excision based on histological characteristics. The primary component of the alveolar mucosa shifts from elastic fibers to collagen fibers. We do not believe there are any limitations at the time of submission. Therefore, we have named this method the alveolar mucosa-switching technique.

## Conclusions

To deepen the sulcus at the shallow vestibule around dental implants in the mandibular molar region, apical positioning of the mobile mucosa (containing enriched elastic fiber) prior to immobile palatal mucosa (containing enriched collagen fiber) grafting is not always necessary. In the histopathological aspect, the replacement of the elastic fiber with collagen fiber is reasonable to establish immobilization of the mucosa. Simple excision of mobile mucosa is desirable to avoid flabby mucosa production and to promote immobile scar tissue formation at the sulcus. We examined this change in the patients histologically and named this method the alveolar mucosa-switching technique.
